# CdS/ZnS core–shell nanocrystal photosensitizers for visible to UV upconversion†
†Electronic supplementary information (ESI) available. See DOI: 10.1039/c7sc01610g



**DOI:** 10.1039/c7sc01610g

**Published:** 2017-05-31

**Authors:** Victor Gray, Pan Xia, Zhiyuan Huang, Emily Moses, Alexander Fast, Dmitry A. Fishman, Valentine I. Vullev, Maria Abrahamsson, Kasper Moth-Poulsen, Ming Lee Tang

**Affiliations:** a Department of Chemistry and Chemical Engineering , Chalmers University of Technology , 412 96 Gothenburg , Sweden; b Materials Science & Engineering Program , University of California, Riverside , 900 University Ave. , Riverside , CA 92521 , USA; c Department of Chemistry , University of California, Riverside , 900 University Ave. , Riverside , CA 92521 , USA . Email: mltang@ucr.edu; d Department of Chemistry , University of California , Irvine , CA 92697 , USA

## Abstract

Herein we report the first example of nanocrystal (NC) sensitized triplet–triplet annihilation based photon upconversion from the visible to ultraviolet (vis-to-UV). Many photocatalyzed reactions, such as water splitting, require UV photons in order to function efficiently. Upconversion is one possible means of extending the usable range of photons into the visible. Vis-to-UV upconversion is achieved with CdS/ZnS core–shell NCs as the sensitizer and 2,5-diphenyloxazole (PPO) as annihilator and emitter. The ZnS shell was crucial in order to achieve any appreciable upconversion. From time resolved photoluminescence and transient absorption measurements we conclude that the ZnS shell affects the NC and triplet energy transfer (TET) from NC to PPO in two distinct ways. Upon ZnS growth the surface traps are passivated thus increasing the TET. The shell, however, also acts as a tunneling barrier for TET, reducing the efficiency. This leads to an optimal shell thickness where the upconversion quantum yield (*Φ*′_UC_) is maximized. Here the maximum *Φ*′_UC_ was determined to be 5.2 ± 0.5% for 4 monolayers of ZnS shell on CdS NCs.

## Abstract 2

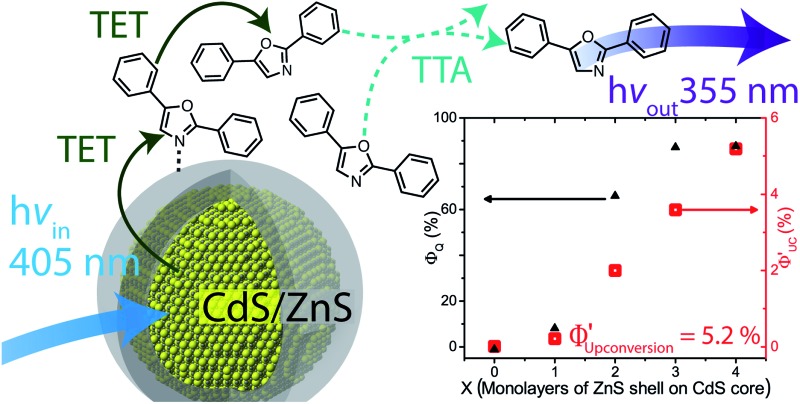
Herein we report the first example of nanocrystal (NC) sensitized triplet–triplet annihilation based photon upconversion from the visible to ultraviolet (vis-to-UV).

## 1 Introduction

It remains a challenge to utilize visible light directly in photocatalysis for the production of solar fuels. Since Honda and Fujishima's pioneering work,^1^ it has been shown that wide bandgap semiconductors like titania^2^ or NiO/NaTaO_3_:La in combination with ultra-violet (UV) radiation can perform efficient chemical transformations. For example, combining NiO/NaTaO_3_:La with 270 nm light results in an extremely high quantum yield of 56% for water splitting.^3^ Various methods to extend the response of these materials to the entire solar spectrum have focused on extending the absorption of the semiconductor, mostly without a commensurate increase in photocatalytic efficiency, due to the introduction of trap states that compromise carrier mobility.^4,5^ The record thus far is a solid solution of GaN/ZnO that can absorb visible light shorter than 500 nm, and split water with a quantum yield >3%. These photocatalysts by Domen and co-workers remain the state of the art in the field.^6–8^

It may be possible to extend the response of photocatalysts to the entire solar spectrum, by using semiconductor nanocrystals (NCs) to absorb the low energy photons, and transferring them to molecular triplet states. With their high absorption coefficients, semiconductor NCs or quantum dots (QDs) have been employed as light absorbers in photovoltaic (PV) cells^9^ and photocatalytic H_2_ production^10,11^ as well as labels for biosensing and imaging.^12^ It has recently been shown that semiconductor NCs can donate^13,14^ and accept^15,16^ triplet excitons to and from molecular species, respectively. Thus semiconductor NCs can be used as triplet sensitizers for triplet–triplet annihilation (TTA) based photon upconversion.^13,14,17–21^ By enabling the use of sub-bandgap photons, TTA photon upconversion can potentially extend the absorption of light harvesting devices,^22,23^ leading to improved efficiencies.^24–35^ For example, in commercially relevant solar cells, up to 34% of the photons from the Sun are wasted in PV platforms where the light absorber has band gaps above 1.1 eV. Therefore, sensitizers capable of efficiently absorbing NIR photons for improving PV efficiency, or for subsequent upconversion to UV photons for photocatalysis are sought after. Indeed, efficient TTA photon upconversion of NIR photons to visible (vis) has been achieved at sub-solar excitation intensities by using NCs as triplet sensitizers.^20,36^ NC based triplet sensitizers are particularly interesting in this aspect as they have high molar absorptivities, good photostability and size-tunable optical profiles.^37,38^ However, to the best of our knowledge, there has been no report of NC sensitized triplet–triplet annihilation based photon upconversion from the vis-to-UV. It is not trivial to develop NCs for efficient triplet sensitization since there is a lot left to unravel regarding the mechanism for efficient triplet energy transfer from NCs to organic molecules. Furthermore, even in purely molecular TTA systems vis-to-UV upconversion is scarce,^39–41^ as suitable annihilator–sensitizer pairs are elusive. This is because the ideal annihilator has to fulfill two requirements: a high fluorescence QY, and a first triplet excited state, T_1_, slightly higher in energy than twice the first singlet excited state, S_1_, *i.e.* 2 × *E*(T_1_) > *E*(S_1_). UV emitters, however, have relatively small exchange energies compared to their more conjugated counterparts. This translates into a relatively high T_1_ compared to S_1_. Since the sensitizer S_1_ and T_1_ both must lie between the annihilator S_1_ and T_1_, it is difficult to find molecular triplet donors that can sensitize these states to achieve efficient photon upconversion. The difficulty in predicting excited state properties, like intersystem crossing efficiencies, makes designing triplet sensitizers with high extinction coefficients, high triplet yields and suitable triplet energies challenging.^42^ In contrast, the exchange energy is on the order of meV for quantum confined semiconductor nanocrystal sensitizers, hence thermodynamic restrictions are relaxed for this class of materials as triplet exciton donors.^43,44^

Herein we demonstrate the first example of vis-to-UV photon upconversion with quantum yields of up to 5.2% utilizing a hybrid organic–inorganic system with a series of CdS/ZnS core–shell nanocrystals as sensitizers. We find that a ZnS shell on CdS cores of 3.6–4.3 nm in diameter is necessary for any appreciable upconversion. Using transient absorption spectroscopy and photoluminescence (PL) lifetime measurements, we show that the ZnS shell removes the trap states on the core, while facilitating triplet energy transfer to molecular annihilators. The optimal shell-thickness occurred at 4 monolayers, corresponding to ∼1.2 nm of ZnS, which is surprisingly thick considering the shell is a tunneling barrier for triplet excitons. We found, unexpectedly, that a transmitter ligand did not enhance the photon upconversion quantum yields. Typically, in these hybrid photon upconversion platforms, a transmitter ligand can increase the upconversion quantum yield by up to 3 orders of magnitude by introducing an energy cascade and improving orbital overlap between the NC donor and the molecular acceptor.^13,14^ This work shows that energy transfer between nanocrystals and conjugated organic molecules is very sensitive to the atomic and molecular details at this hybrid interface.

## 2 Results and discussion

**Fig. 1:**
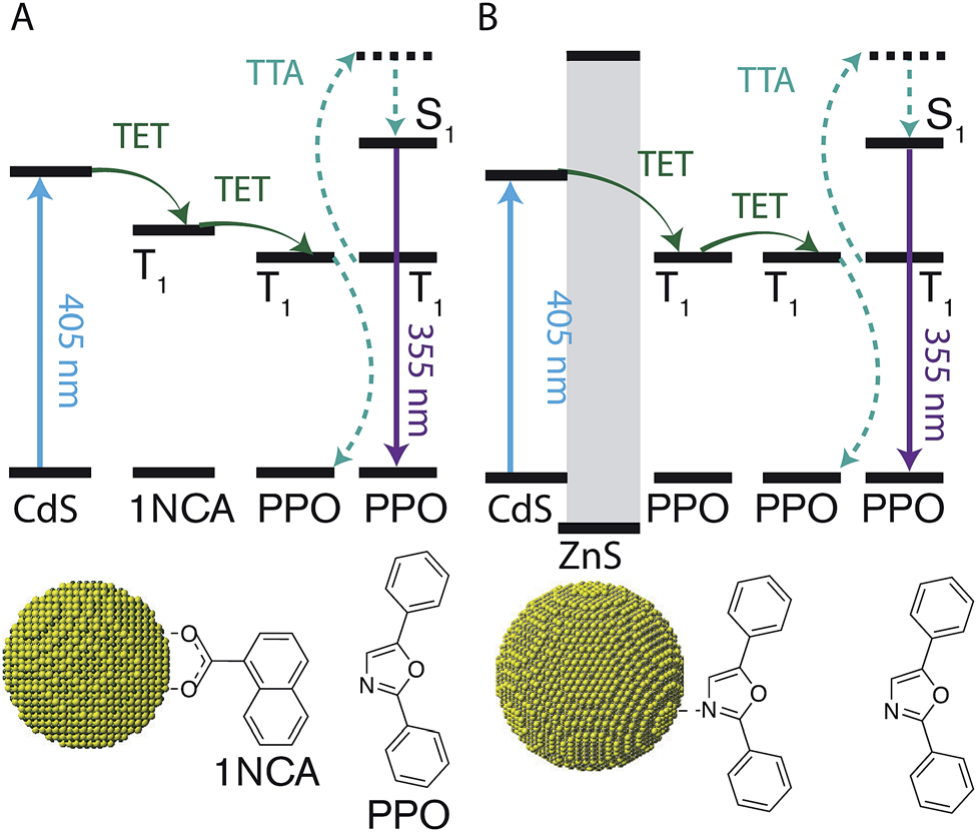
Schematic illustration of the studied triplet–triplet annihilation (TTA) based photon upconversion systems. (A) Triplet energy transfer from CdS nanocrystals (NCs) to a bound 1-naphthoic acid (1NCA) molecule, followed by triplet energy transfer (TET) to the annihilator 2,5-diphenyloxazole (PPO). PPO undergoes TTA with another triplet excited PPO upconverting visible light to UV photons. (B) Like in (A) but for CdS/ZnS core–shell NCs with PPO as both the bound transmitter ligand and free annihilator.

A scheme of all the components involved in the energy transfer during photon upconversion is presented in Fig. 1. The energy offsets of the three components are also illustrated. In this study we employed the scintillator 2,5-diphenyloxazole (PPO) as the annihilator. PPO emits UV photons with an emission maxima of 355 nm (3.49 eV) in hexane and has a high fluorescence quantum yield close to unity.^45^ The first excited triplet state, T_1_, of PPO lies in the range of 2.3–3.0 eV,^39,46^ corresponding to the visible wavelength range of 400–540 nm. Therefore, CdS NCs with the appropriate band-gap slightly higher in energy than this T_1_ state were used as photosensitizers for PPO.

### 2.1 Nanocrystal synthesis and characterization

Two sets of CdS NCs were synthesized from CdO and elemental sulfur *via* the hot injection method,^47^ yielding NCs with a diameter of 3.6 nm and 4.3 nm, according to the first exciton absorption maxima at 405 nm and 427 nm respectively.^48^ The 3.6 nm and 4.3 nm CdS cores were subsequently coated with 1–4 and 3–5 monolayers of ZnS respectively using zinc-diethyldithiocarbamate as the precursor, similar to the procedure reported by Chen *et al.* ^49^ In the rest of the text, shell thickness for the CdS/ZnS core–shell NCs will be indicated by *X*ML, where *X* = 0, 1, 2, 3, 4 and 5 and ML = monolayers.

**Fig. 2:**
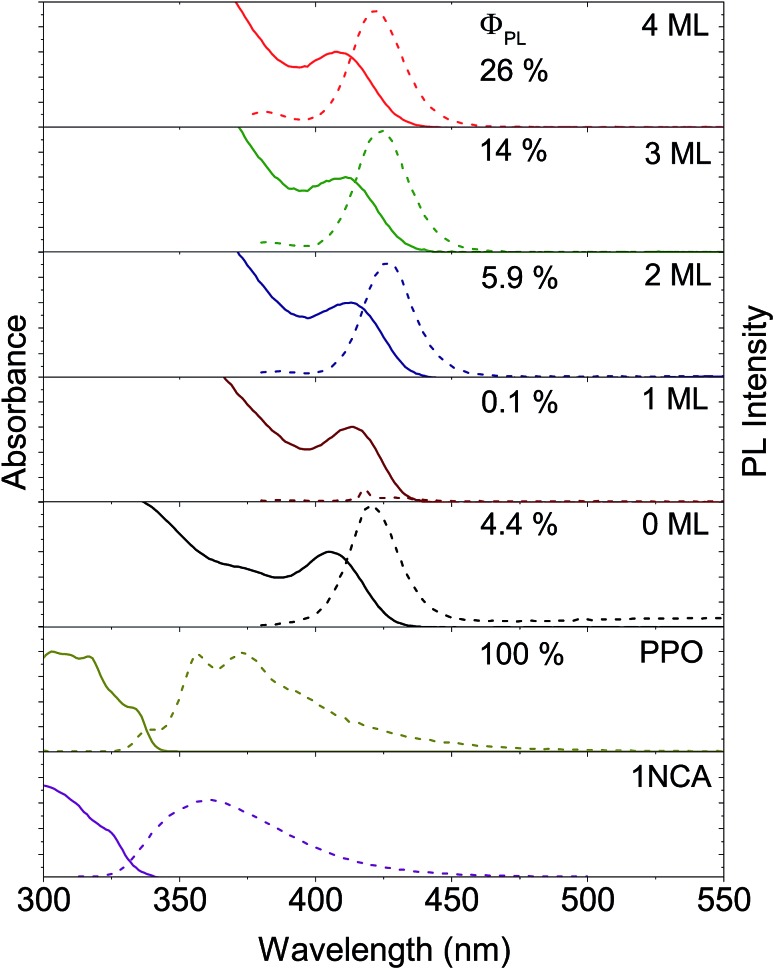
Absorption (solid) and emission (dashed) spectra of 1-naphthoic acid (1NCA), 2,5-diphenyloxazole (PPO), 3.6 nm diameter CdS core only nanocrystals (0ML) and CdS/ZnS core–shell nanocrystals with 1–4 monolayers (1–4ML). Displayed are also the photoluminescence quantum yields (*Φ*_PL_).

Growth of the first ZnS monolayer (1ML) red-shifts the excitonic absorption maxima about 9 nm compared to the CdS core. Table 1 lists the optical properties for the core–shell NCs derived from the 3.6 nm diameter CdS cores. This bathochromic shift is expected for cation terminated NCs as the addition of an outer layer of sulfide anions delocalizes the exciton compared to the original core.^38^ Further growth of the ZnS shell results in a gradual blue-shift (Table 1, Fig. 2 and S1†), suggesting the formation of an alloy.^49,50^ TEM images of the 4.3 nm CdS core and 3–5ML core–shell NCs can be found in the ESI, Fig. S2.†

**Table 1:** The absorption maxima, Abs_max_; emission maxima, Em_max_; photoluminescence quantum yield, *Φ*_PL_; and amplitude weighted average photoluminescence lifetimes of the 3.6 nm diameter CdS nanocrystals (NC) with ZnS shells of different monolayer (ML) thicknesses. *k*_r_ is the radiative rate of the NC and *k*_nr_ is the non-radiative rate. *τ*_0_ and *τ*_PPO_ indicate the NC lifetimes without and with the PPO annihilator^*a*^

NC	Abs_max_ (nm)	Em_max_ (nm)	*Φ*_PL_ (%)	*k*_r_ ^*b*^ (s^–1^)	*k*_nr_ ^*b*^ (s^–1^)	*τ*_0_ (ns)	*τ*_PPO_ (ns)
0ML	405	421	4.4	5.3 × 10^5^	0.1 × 10^8^	16.9	17.1
1ML	414	418	0.1	4.2 × 10^5^	7.0 × 10^8^	0.25	0.23
2ML	413	427	5.9	1.1 × 10^7^	1.8 × 10^8^	1.35	0.46
3ML	411	425	14	1.6 × 10^7^	1.0 × 10^8^	4.11	0.53
4ML	407	422	26	2.8 × 10^7^	0.8 × 10^8^	5.80	0.72

^*a*^All measurements were done in hexane.

^*b*^ *k*_r_ and *k*_nr_ are calculated from the intensity weighted average lifetimes *τ̄* as described in the ESI.

Photoluminescence from both the band-edge and trap states is observed (Fig. 2 and S1†) for both CdS core NCs. For the 3.6 nm CdS core the band-edge emission is observed as a sharp peak at about 420 nm and the trap state emission as a broad peak in the range of 450–650 nm. The latter is ascribed to surface-based trap states arising from sulfur vacancies.^51^ This trap-state emission was completely removed with the growth of more than 2 monolayers of ZnS shell on the CdS core, indicating that shell growth passivates the surface traps. This results in higher photoluminescence quantum yields (*Φ*_PL_), up to 26% for the 4ML core–shell NCs, compared to 4.4% for the 3.6 nm CdS core, as summarized in Fig. 2 and Table 1. For 1ML core–shell NCs, almost no emission could be detected, possibly due to an incomplete shell, in line with previous results.^49,52^

### 2.2 Visible to UV upconversion

Much like triplet energy transfer (TET) between molecules in solution, which is governed by the Dexter exchange mechanism and limited by diffusion, the TET here depends on both the acceptor concentration and sensitizer lifetime. Thus, a long triplet lifetime of the sensitizer is desired to achieve high TET efficiencies. Typically a molecular triplet sensitizer has a lifetime of 10–100 s of μs. As NCs generally have shorter lifetimes, a transmitter ligand with a long-lived triplet state is helpful.^13,14,53^ Carboxylic acid functionalized ligands, such as 9-anthracenecarboxylic acid (9ACA) and 5-tetracenecarboxylic acid (5CT) are effective triplet transmitters for CdSe and PbS(Se) NCs, respectively.^13,14,19,54^ The carboxylic functionality allows for binding to chalcogenide nanoparticles without quenching the NC PL and the fused aromatic system has a triplet energy level suitable for it to act as an acceptor to enable triplet energy transfer from NC to ligand. Therefore our first choice of transmitter ligand was a molecule with a carboxylic acid binding group and a naphthalene core in the form of 1-naphthoic acid (1NCA) as its triplet energy is about 2.48 eV (corresponding to 500 nm),^55^ which is within the range of the reported T_1_ levels of the PPO annihilator (400–540 nm).

For comparison between photon upconversion systems the upconversion quantum yield (*Φ*_UC_) is typically the main figure of merit. It can be determined relative to a standard according to eqn (1),1
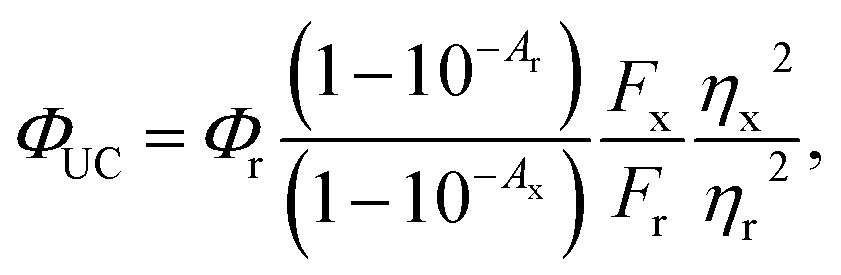
where *A*_i_ is the absorption at the excitation wavelength, *F*_i_ is the integrated emission and *η*_i_ is the refractive index of the solvent, subscripts x and r denote the sample and reference, respectively. It is important to note that in this definition the maximum upconversion quantum yield is 50% as two low energy photons are consumed to produce one photon of higher energy. Often the upconversion quantum yield is normalized to a maximum of 100% by multiplying eqn (1) with a factor 2. To avoid confusion we here denote this normalized value as *Φ*′_UC_ = 2*Φ*_UC_.

An upconverting system consisting of CdS NCs (core only, 0ML) as photosensitizer, 1NCA as transmitter ligand bound to the NC and PPO as annihilator yielded only low *Φ*′_UC_ of ∼0.8%. No photon upconversion was detected without the 1NCA transmitter ligand bound to the CdS NCs, nor was it observed without the CdS NCs. Furthermore, CdSe NC sensitizers of various sizes (absorption maxima in the range of 480–520 nm) did not afford any detectable upconversion.

**Fig. 3:**
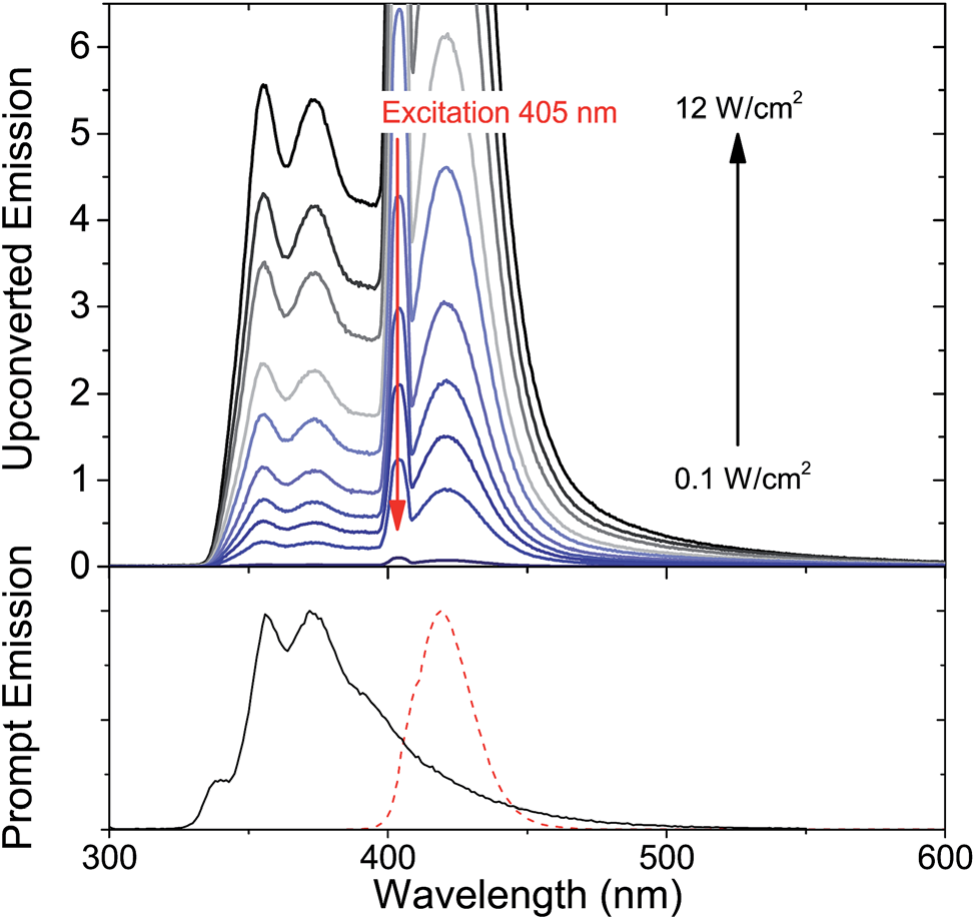
(Top) Upconverted emission from 2,5-diphenyloxazole (PPO) in hexane, sensitized by 3.6 nm CdS core nanocrystals with 4 monolayers (4ML) of ZnS shell, excited at 405 nm. Excitation power density is varied from 0.1 W cm^–2^ to 12 W cm^–2^. For clarity only spectra with excitation densities >1.3 W cm^–2^ are displayed. (Bottom) Prompt emission of PPO (solid) and 4ML (dashed) ZnS on 3.6 nm CdS core for comparison.

Since it is well known that core–shell NCs have fewer surface trap-states compared to core only NC^10,11,21,37,38^ we expected CdS/ZnS core–shell NCs to be more efficient for photosensitization. The entire series of CdS/ZnS core/shell NCs with 1–4ML of ZnS shell did not require the transmitter ligand 1NCA to achieve detectable upconversion. In fact, there was no difference in the upconversion quantum yield (*Φ*′_UC_) with and without the 1NCA transmitter. This is most likely due to the possibility of PPO binding to the ZnS shell through the oxazole nitrogen and a mismatch between the triplet energy levels of 1NCA and PPO. Upon 405 nm excitation of 4ML core–shell NCs (1 μM) with PPO (5.7 mM in hexane) upconverted PPO emission was readily detected, as seen in Fig. 3, and *Φ*′_UC_ was determined to 5.2 ± 0.5% at 7.1 W cm^–2^, in the linear excitation regime (Fig. S3†). The linear regime is reached at a rather high excitation power density (>1 W cm^–2^), Fig. S3,† and for practical applications it must be reduced.

**Fig. 4:**
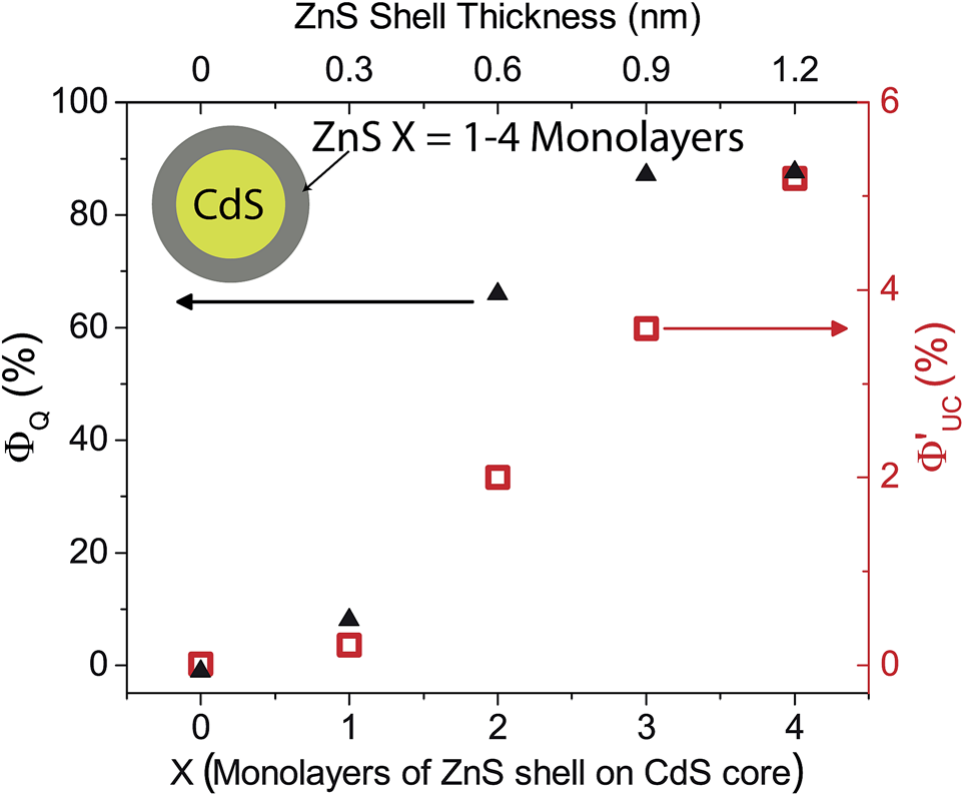
Photoluminescence quenching efficiency (*Φ*_Q_, black triangles) of 3.6 nm nanocrystals by 5.7 mM 2,5-diphenyloxazole (PPO) in hexane as a function of ZnS shell thickness. The upconversion quantum yield (*Φ*′_UC_, red squares) for the same samples is shown, upon 405 nm excitation at 7.1 W cm^–2^.

*Φ*′_UC_ increases with ZnS shell thickness up to 4 monolayers. Further ZnS shell growth leads to a decrease in the photon upconversion quantum yield. This trend was observed for both sets of CdS/ZnS core–shell NCs. Data for the 3.6 nm diameter series is summarized in Fig. 4, while that for the 4.3 nm diameter series is in Fig. S4.† It is somewhat surprising that *Φ*′_UC_ initially increases with the layer thickness as it has been reported previously that both CdS and ZnS shells acts as a tunneling barrier for hole transport from CdSe NCs to surface bound ligands.^56,57^ Hence, after passivation of the trap states, the ZnS shell is expected to decrease TET from the CdS core to the molecular acceptors. Charge and energy transfer are dictated by the energy offset between the donor and acceptor as well as the tunneling barrier. Comparing 0ML core only CdS NCs and 4ML CdS/ZnS core–shell NCs, both have similar absorption maxima (405 nm and 407 nm, respectively). Therefore the driving force for TET is the same for both NC sensitizers, and only depends on the energetics of the triplet state of the molecular acceptor. TET is downhill by ∼0.5 eV and ∼0.7 eV for 1NCA and PPO respectively. It should, however, be noted that the spread of reported PPO triplet energy levels in the literature is wide and the driving force for PPO could, in fact, be lower than 1NCA.

### 2.3 Effect of ZnS shell

To increase our understanding of the effect of the ZnS shell and how it affects TET and therefore photon upconversion, we performed time-resolved PL (TRPL) of the NCs both with and without PPO. NCs often show multi-exponential decays, even after shell growth^11,58,59^ and a three exponential fit was required to achieve a satisfactory fit to the data. Both amplitude and intensity weighted average lifetimes, *τ* and *τ̄* respectively, were extracted from the fits. *τ* values were used to calculate the quenching efficiencies whereas the radiative decay rates *k*_r_ were obtained from *τ̄*, as described in the ESI.† Fig. S5 and S6† show the decays and fits. In Table 1 the amplitude weighted average lifetime *τ* is listed. A similar trend is observed for *τ̄* and can be found in the ESI, Table S1.† As can be seen in Table 1, 2–4 monolayers of ZnS shell increase the radiative decay rates by an order of magnitude with respect to the shell. In a type I core–shell NC, both the electron and hole are confined to the core due to its band offsets with respect to the shell, thus increasing the radiative rate due to enhanced wavefunction overlap. A type I structure is expected for CdS/ZnS core–shell NCs since the bandgap of bulk CdS lies within the bandgap of bulk ZnS.^38,60^ The ZnS shell eliminates the trap state emission to the red of the excitonic absorption maxima seen in the CdS NCs (Fig. 2 and S1†). This passivation of surface trap states explains the increase in *Φ*_PL_ with respect to the CdS core only NCs.^60^

With a thicker shell, however, the tunneling barrier for TET from NC core to the molecular acceptors would increase, leading to a decrease in the TET rate.^56,57^ These two opposing effects on the TET with shell thickness would explain why there is an optimum shell thickness where the maximum *Φ*′_UC_ is observed.

### 2.4 Triplet energy transfer from NC to PPO

**Fig. 5:**
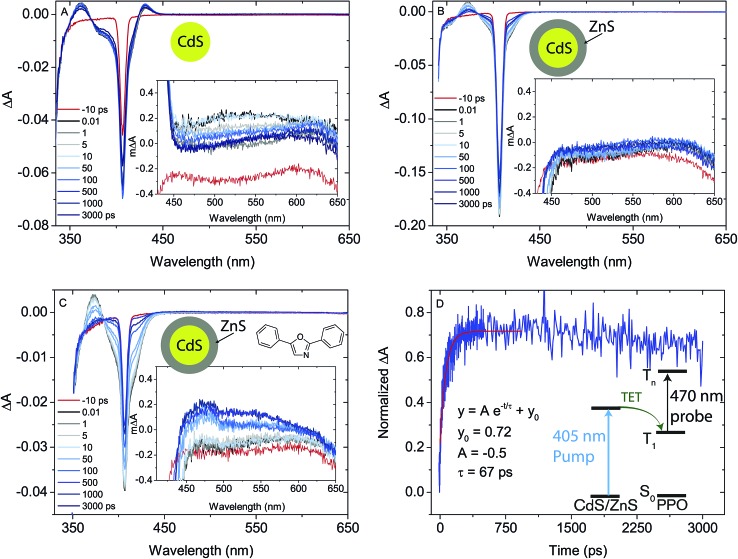
Transient absorption of nanocrystals (NCs) with 3.6 nm diameter CdS core. (A) CdS core only NCs, (B) CdS/ZnS core–shell NCs with 4 monolayers (4ML) and (C) CdS/ZnS core–shell NCs 4ML with 2,5-diphenyloxazole (PPO). Inset in (A–C) shows the region 450–650 nm. (D) The rise of the signal at 470 nm in (C) (blue), corresponding to the PPO T_1_–T_*n*_ absorption and monoexponential fit (red). Also shown in (D) are the fitting parameters and an energy diagram of the T_1_–T_*n*_ transition of PPO after sensitization by CdS/ZnS.

From the PL lifetimes the quenching efficiency (*Φ*_Q_) of PPO on the NCs can be calculated according to eqn (2),2
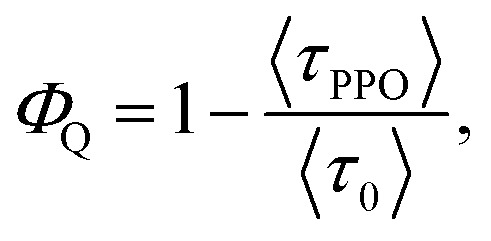
where *τ*_PPO_ and *τ*_0_ are the amplitude weighted averaged PL lifetimes of the NCs with and without PPO, respectively. The quenching efficiency correlates relatively well with *Φ*′_UC_ for both batches of core–shell NC, as seen in Fig. 4 and S4,† indicating that the emissive states are quenched due to TET from the NC to PPO. The high quenching efficiency, close to 90%, would indicate that TET from core–shell NC to ligand is very efficient. To further understand the TET from NC to PPO and the effect of the ZnS shell, femtosecond transient absorption (fs-TA) spectroscopy was performed on the 3.6 nm diameter CdS core NCs, with and without PPO, and the same NCs with 4ML of ZnS shell, again in the presence and absence of PPO (5.7 mM in hexane), Fig. 5 and S7.† Excitation intensities were chosen to ensure no multi-photon annihilation occurred in the NCs, see Fig. S8.† Core only CdS NCs (0ML) with and without PPO showed similar features and dynamics, indicating that the ligand PPO does not interact with the excited state of the NC in the 3 ns window, explaining the low upconversion quantum yields from these samples. This is in line with the time-resolved PL data for the CdS core, where no change in the NC's PL lifetime was observed in the presence of PPO (Fig. S5 and S6†). Comparing the TA spectra of 0ML CdS NCs and 4ML CdS/ZnS core–shell NCs there are some notable differences. In the CdS core only samples (0ML) there is a positive absorption feature to the red of the ground-state bleach, at ∼430 nm, which is absent for core–shell NCs 4ML. This positive feature is known to arise from the Stark effect induced by trapped carriers,^61–65^ indicating the presence of trap-states in the CdS NCs. This excited state absorption is absent in the 4ML core–shell CdS/ZnS NCs and indicates successful passivation of the trap-states in these heterostructures.

Furthermore, for 4ML CdS/ZnS core–shell NCs with PPO, there is also a new positive feature at 450–600 nm, ascribed to the T_1_–T_*n*_ absorption of PPO,^66–68^ verifying TET from the core–shell NC to PPO. The rise of the T_1_–T_*n*_ feature can be fit to a monoexponential with a rise-time of 67 ps, corresponding to a rate constant of 14.9 × 10^9^ s^–1^, an order of magnitude faster than that reported previously for TET from CdSe NCs to 9-anthracenecarboxylic acid.^14^ The fast TET is in agreement with the efficient PL quenching of the NC by PPO.

The upconversion quantum yield depends on the efficiencies of the processes involved and can be described by eqn (3),

3 *Φ*′_UC_ = 2*Φ*_ISC_*Φ*_TET_*Φ*_TTA_,

where *Φ*_ISC_, *Φ*_TET_ and *Φ*_TTA_ are the efficiencies for intersystem crossing within the sensitizer, triplet energy transfer from the sensitizer to the annihilator and triplet–triplet annihilation between two annihilators, respectively. Our experiments indicate that the TET is efficient, close to 90%, meaning either the intersystem crossing or triplet–triplet annihilation efficiencies limit the overall upconversion efficiency. In NCs the singlet–triplet exchange energy is small, smaller than *kT* at room temperature, thus singlet–triplet mixing is large.^43,44^ Therefore one would expect the triplet formation in such a case to be efficient. This leaves the limiting step in photon upconversion to be triplet–triplet annihilation.

Triplet–triplet annihilation can be limited by spin-statistics if the second triplet state (T_2_) and the first quintet state (Q_1_) are lower in energy than 2 × *E*(T_1_), resulting in *Φ*′_UC_ less than 11%.^69^ Furthermore, we have recently shown that there are other loss factors influencing the TTA process.^70^ PPO has been used previously as an annihilator with two different molecular sensitizers and the reported *Φ*′_UC_ values are low, <1%.^39,40^ This could indicate that PPO does have intrinsic limitations as the annihilator, possibly originating from its high T_1_ energy. The relatively high T_1_ energy also limits the efficiency of energy conversion in the present system to blue-to-UV upconversion. Finding a better annihilator, with 2 × *E*(T_1_) only slightly exceeding *E*(S_1_), a larger part of the visible spectrum could be converted with a higher efficiency. This is currently under investigation in our lab. With the efficient sensitization by core–shell NCs demonstrated here, we have, however, increased the upconversion efficiency (*Φ*′_UC_) by a factor of 5 compared to the all-molecular systems employing PPO as the annihilator.

## 3 Conclusion

Herein we demonstrate the first example of visible to UV upconversion using NC sensitizers for TTA-based photon upconversion. This work extends the broad range of wavelengths possible for photon upconversion based on triplet fusion sensitized by NCs from the NIR and visible into the UV. Sensitization of the annihilator PPO was achieved efficiently by CdS/ZnS core–shell NCs with the optimal shell thickness of 4 monolayers. The effect of the ZnS shell is twofold. First the surface traps of the CdS core are removed, secondly the large bandgap shell acts as a tunneling barrier for TET, as observed for hole and electron transfer.^56,57^ The former has a positive effect on TET and consequently *Φ*′_UC_, the latter on the other hand decreases TET as the shell thickness increases. Our data suggests that there is an optimal thickness where these two effects are balanced, resulting in the most efficient TET and *Φ*′_UC_. These results contribute to the ongoing investigation of TET from NCs to organic ligands. In particular, it improves the understanding of the requirements for efficient triplet energy transfer (TET) between semiconductor NCs and organic molecules necessary for future design of efficient triplet sensitizer NCs.

## 4 Experimental section

### 4.1 Chemicals

Zinc diethyldithiocarbamate (Zn(DDTC)_2_, 99.0%) was purchased from TCI America, washed with water and dried under vacuum for 8 h before use. Cadmium oxide (CdO, 99.99%), 2,5-diphenyloxazole (PPO, for scintillation), 1-naphthoic acid (1NCA, 96%) were all purchased from Sigma-Aldrich. Sulfur powder (99%) was purchased from Strem Chemicals, oleic acid (90%) was purchased from Alfa Aesar. 1-Octadecene (ODE, tech. grade 90%) was purchased from Acros Organics. All chemicals were used without further purification if not stated otherwise.

### 4.2 Synthesis of CdS core NCs

Synthesis of CdS cores was based on the procedure by Li *et al.* ^47^ CdO, 257 mg (2 mmol), 6 ml oleic acid and 15.8 ml ODE was mixed in a 50 ml, three-necked flask equipped with condenser and temperature controller. The reaction mixture was degassed under vacuum at 110 °C for 1 hour. The mixture was then stirred and heated to 260 °C under argon, until it turned clear and colorless. 32 mg of sulfur powder in 3 ml ODE was prepared in a glove box and sonicated. The sulfur precursor was injected to the reaction mixture at 240 °C (260 °C for larger NCs), stirred for 25 s, then cooled with compressed air to room temperature yielding a pale, colorless reaction mixture with NC absorption maxima at 405 nm (427 nm for larger NCs). The particles were purified in an Ar(g) glove box with extraction by methanol, hexane and butylamine (1 : 0.7 : 0.04, v/v/v). The organic fraction was collected and extracted again with methanol and hexane (1 : 2, v/v). The organic fraction was then precipitated using acetone with a few drops of butylamine. After re-dispersing the NC pellet in hexane, the NCs were precipitated twice more with acetone and stored in hexane at micromolar concentrations.

### 4.3 Growth of ZnS shell

Using the CdS core, synthesized as described above, the ZnS shells were grown according to the procedure by Chen *et al.* ^49^ Zn(DDTC)_2_ was dissolved in oleylamine to prepare a 0.1 M solution. In the glove box, 0.13 μmol CdS core in ODE was dissolved in 8.5 ml ODE in a 27 ml vial. For the first monolayer, 0.47 ml Zn(DDTC)_2_ (0.1 M) was added and the mixture was stirred and heated to 185 °C for 25 min. A second portion of Zn(DDTC)_2_ was added at 50 °C to grow the second monolayer, followed by heating to 185 °C for 30 min. This procedure was repeated until the number of desired monolayers was achieved. The amount of Zn(DDTC)_2_ needed for each layer was calculated as described in the ESI.†

The core–shell NCs were purified by extraction with methanol and hexane (6 : 1, v/v) twice, followed by precipitation of the core–shell NCs by addition of acetone to the organic phase. The particles were re-dissolved in hexane and precipitated twice more with acetone and stored in hexane at micromolar concentrations.

### 4.4 Steady-state absorption, photoluminescence and upconversion experiments

Steady-state UV/vis absorption was recorded in 10 mm path length quartz cuvettes on a Jasco 670 spectrophotometer. Photoluminescence spectra were recorded on a Horiba SpexFluorolog 3 fluorescence spectrophotometer using a right-angle sample geometry. Photoluminescence quantum yields, *Φ*_PL_, were determined relative to 9,10-diphenylanthracene in hexane (*Φ*_PL_ = 0.95 (ref. 71)).

Upconverted emission was detected on an Ocean Optics Maya2000 Pro spectrometer. Excitation of the upconversion samples was a 405 nm cw OBIS laser with a beam radius of 0.2 mm. Samples were prepared in a glove box by adding 15 nmol core–shell NCs (10–80 μl) to 100 μl THF and precipitated with 1 ml acetone, then half of the particles were re-dissolved in 0.7 ml PPO stock solution (5.7 mM) for upconversion experiments and the other half was re-dissolved in 3 ml hexane for PL measurements. Samples were sealed in quartz cuvettes using Teflon backed screw caps to ensure an air-free solution. Herein we have chosen to report the normalized upconversion quantum yield (*Φ*′_UC_ = 2*Φ*_UC_) with the theoretical maximum upconversion quantum yield being 100%. Reported *Φ*′_UC_ values are the average of at least 2 measurements. The upconversion quantum yield *Φ*′_UC_ was determined relative to perylene in degassed ethanol (*Φ*_PL_ = 92% (ref. 72)).

### 4.5 Transient absorption measurements

Transient absorption were performed using the Ti:sapphire chirped pulse amplified system Spitfire-ACE (Spectra Physics, Newport Corp, 6 mJ@800 nm, 100 fs, 1 kHz) and Transient Absorption Spectrometer (TAS, Newport Corp.). The amplifier system is pumped using an Empower® Q-switched DPSS laser and seeded by a Mai Tai SP oscillator (90 fs, 80 MHz). The outcome of the regenerative amplifier was split in multiple paths. One beam was coupled to optical parametric amplifiers (OPA, Lightconversion TOPAS™) to generate a pump pulse at 405 nm (100 fs pulse duration). A small portion of the fundamental 800 nm beam was picked off and sent through a delay line and then focused into a 2 mm CaF_2_ crystal to generate a white light continuum. The generated supercontinuum is then focused onto the sample and overlapped with the pump beam. The transient spectra were detected with fiber-coupled CCD-based monochromator (Oriel, Newport). The excitation power was set to 200 μW, which was below the multi-photon annihilation regime, see ESI† for further details.

### 4.6 Time-correlated single photon counting (TCSPC)

Photoluminescence lifetimes were recorded on a JY Horiba Fluorolog time-correlated single photon counting setup, with a NANOLed laser diode with peak wavelength at 406 nm and a repetition rate of 10 MHz. For detection a 512 channel PMT (TBX Picosecond Photon Detection Module from JY Horiba) was used and a count rate of <1% was maintained. Fits were made to three exponential decays with an *y*-offset and the amplitude averaged lifetime *τ* was extracted from the fits, see the ESI† for further details.

### 4.7 TEM imaging

TEM images were recorded on a FEI Titan Themis 300 with an X-FEG electron gun.
