# Supplementary material for: CdS/ZnS core–shell nanocrystal photosensitizers for visible to UV upconversion
Source: Chem Sci. 2017 May 31;8(8):5488–96. doi: 10.1039/c7sc01610g (PMC5613741; doi:10.1039/c7sc01610g)
Supplement: Supplementary file 1 [file SC-008-C7SC01610G-s001.pdf]

Electronic Supplementary Material (ESI) for Chemical Science.
This journal is © The Royal Society of Chemistry 2017

Electronic Supplementary Information for: CdS/ZnS core-shell nanocrystal photosensitizers for Visible to UV upconversion

Victor Gray,† Pan Xia,‡ Zhiyuan Huang,¶ Emily Moses,‡ Alexander Fast,§ Dmitry A. Fishman,§ Valentine I. Vullev,¶ Maria Abrahamsson,† Kasper Moth-Poulsen,† and Ming Lee Tang*,¶

Department of Chemistry and Chemical Engineering, Chalmers University of Technology, Gothenburg, Sweden, Materials Science & Engineering Program, University of California, Riverside, 900 University Ave., Riverside, CA, 92521, USA, Department of Chemistry, University of California, Riverside, 900 University Ave., Riverside, CA, 92521, USA, and Department of Chemistry, University of California, Irvine, CA, 92697, USA

E-mail: mltang@ucr.edu

*To whom correspondence should be addressed

†Department of Chemistry and Chemical Engineering, Chalmers University of Technology, Gothenburg, Sweden

‡Materials Science & Engineering Program, University of California, Riverside, 900 University Ave., Riverside, CA, 92521, USA

¶Department of Chemistry, University of California, Riverside, 900 University Ave., Riverside, CA, 92521, USA

§Department of Chemistry, University of California, Irvine, CA, 92697, USA

Ligand exchange for CdS and 1NCA

Replacing a fraction of the native OA ligand with 1NCA was done by stirring 10 μM CdS in 150 μl Hexane/THF (1:4, v/v) with 1NCA for 40 minutes in an Ar(g) glovebox. It was found that the optimal ration of 1NCA to CdS was 250:1. After ligand exchange the NC were precipitated with acetone and redissolved in hexane with PPO for UC measurements.

Calculations to determine amount of $Zn(DDTC)_2$ needed for shell growth

Calculations for the amount of $Zn(DDTC)_2$ is based on the method used by Chen *et al.*[S1]

The size of CdS core: d_0 = 3.55 nm r_0 = 3.55/2 = 1.77 nm

The amount of CdS particles: $n_1 = 1.3 \text{ x } 10^{-7}$ mol

Lattice constant of ZnS:

For Zinc blende a = 0.5406 nm

Wurtzite a = 0.3814 nm c = 0.6257 nm

The average thickness of one monolayer of ZnS (d):

For Zinc blende $d = \frac{\sqrt{3}}{3}a = \frac{0.5406\times1.732}{3} = 0.312$ nm

For Wurtzite $d = \frac{c}{2} = 0.6257\times0.5 = 0.313$ nm

Here we used d = 0.31 nm

The volume of the first ZnS monolayer:

$r_0 = 1.77$ nm

$r_1 = 1.77+0.31 = 2.08$ nm

$V_1 = 4 \times 3.14 \times \frac{2.083-1.773}{3} = 1.45 \times 10^{-26}$ m^3

The density of ZnS:

For Zinc blende: $\rho = 4.10\times10^{-3}$ $kg/cm^3 = 4.10\times10^6$ g/m^3

Wurtzite $\rho = 4.09\times10^{-3}$ $kg/cm^3 = 4.09\times10^6$ g/m^3

Here, we used, $\rho = 4.1\times10^6$ g/m^3

The mass of the 1st ML ZnS shell in one core/shell particle: m = Volume × Density

$m = 1.45 \times 10^{-26}\ m^3 \times 4.1\times10^6\ g/m^3 = 5.95\times10^{-20}$ g

The amount of ZnS in one core/shell particle: n = m/M.W.

M.W. (Molecular weight) = 97.474 g/mol

$n_2 = \frac{5.95\times10^{-20}}{97.474} = 6.10\times10^{-22}$ mol/particle

The amount of $Zn(DDTC)_2$ precursor for 1ML ZnS growth: $A1 = n_1 \times N \times n_2$

$A_1 = 1.3\times10^{-7}$ mol × (6.025×10^{23} particle/mol) × (6.10×10^{-22} mol/particle) = 4.72×10^{-5} mol

2ML ZnS: $A_2 = A_1\times V_2/V_1 = 0.0472\times\frac{(0.31\times1+r_1)^3-(0.31\times0+r_1)^3}{r_1^3-r_0^3} = 0.064$ mmol

3ML ZnS: $A_3 = A_1 \times V_3/V_1 = 0.0472\times\frac{(0.31\times2+r_1)^3-(0.31\times1+r_1)^3}{r_1^3-r_0^3} = 0.082$ mmol

4ML ZnS: $A_4 = A_1 \times V_4/V_1 = 0.0472\times\frac{(0.31\times3+r_1)^3-(0.31\times2+r_1)^3}{r_1^3-r_0^3} = 0.104$ mmol

3-5 Monolayer CdS/ZnS

3 to 5 ZnS ML were grown on 4.3 nm CdS cores. The photoluminescence quantum yield (Φ_{PL}) increased from 0.9% for the core to 47% for 5ML. Absorption and emission spectra are displayed in Figure S1.

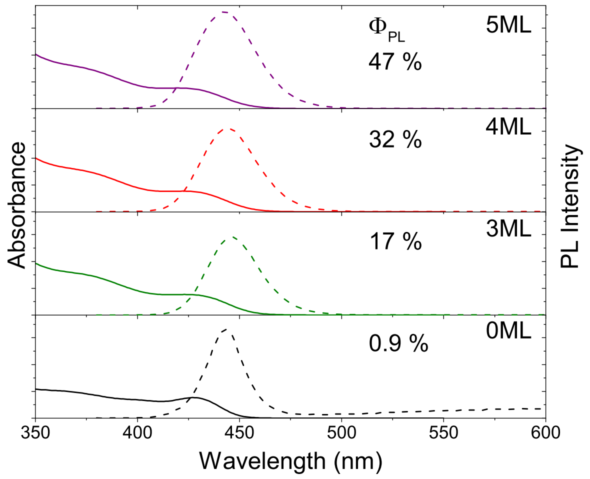

Figure S1: Absorption (solid lined) and emission (dashed lines) spectrum of 4.3 nm diameter CdS nanocrystals (0ML), and CdS/ZnS core-shell nanocrystals with 3-5 monolayers (3ML, 4ML and 5ML, respectively) in hexane.

TEM images

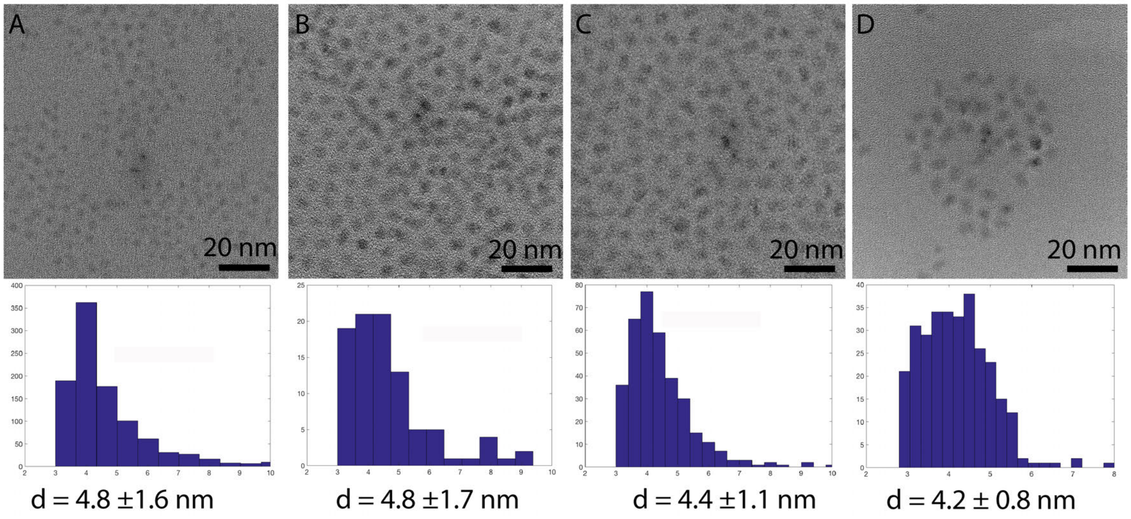

Figure S2: TEM images of synthesized CdS nanocrystals (NCs) and CdS/ZnS NCs with core 4.3 nm and 3-5 monolayers (3ML, 4ML and 5ML respectively). A) CdS core, 0ML. B) 3ML CdS/ZnS. C) 4ML CdS/ZnS. D) 5ML CdS/ZnS. The histograms shows the size distribution of the visible CdS core.

TEM images of the synthesized CdS and CdS/ZnS NCs with 4.3 nm core are shown in Figure S2. Shown are also histograms of the size distribution. However, the low contrast of CdS and in particular ZnS in TEM images makes it difficult to determine the size accurately from these figures, consequently the extracted diameter represents the core-size. In regions where the NCs are close-packed, the average center-to-center distance increases from 4.9±0.6 nm to 8.2±0.9 nm when going from 0 to 5 monolayers. As a shell of 5 monolayers corresponds to an increase in diameter of about 3 nm, this is in good agreement with the observed center-to-center distance increase.

Upconversion Measurements

The upconverted emission as a function of excitation intensity of CdS/ZnS **4ML** NCs with PPO in hexane is displayed in Figure S3. Upconversion quantum yields (Φ'_{UC}) measured for core shell NCs with 4.3 nm cores are shown in Figure S4. After 4 monolayers Φ'_{UC} starts decreasing. Reported Φ' values are average of minimum 2 measurements.

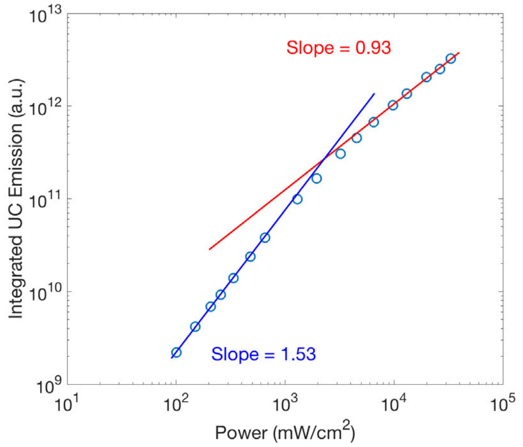

Figure S3: Upconverted emission from 2,5-diphenyloxazole (PPO) after sensitization by a 4 monolayer (**4ML**) CdS/ZnS nanocrystal (NC) with 3.6 nm diameter CdS core as a function of excitation intensity at 405 nm, in hexane.

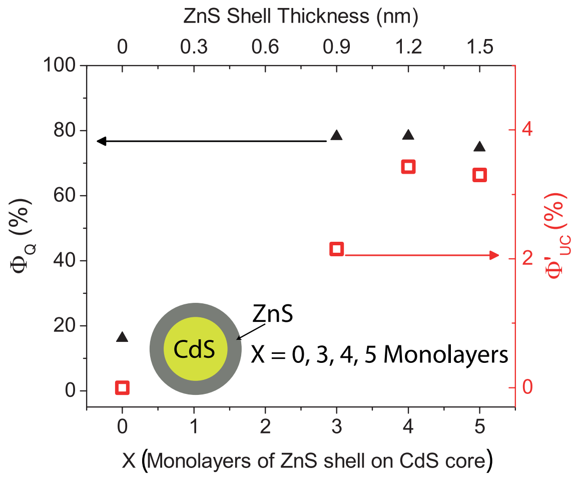

Figure S4: Photoluminescence quenching efficiency (Φ_Q, black triangles) of 4.3 nm nanocrystals (NCs) by 5.7 mM 2,5-diphenyloxazole (PPO) in hexane as a function of ZnS shell thickness. The upconversion quantum yield (Φ'_{UC}, red squares) for the same samples is shown, upon 405 nm excitation at 7.1 W cm^{-2}.

Time-Correlated Single Photon Counting, TCSPC

Nanocrystal PL decays are often multi-exponential and therefore difficult to fit. We chose to use a three exponential decay model with a y-offset to account for life-times longer than the time-range recorded. Attempts using a 4-exponential model did not improve the fits significantly. The decays were deconvoluted from the instrument response function and the goodness of fit was judge mainly by examining the residuals as the low number of channels (512) and deviations from the fits at longer times resulted in relatively high χ^2 values. The decays and fitted functions are displayed in Figures S5-S6 and the determined lifetimes and amplitudes are listed in Table S1.

For multi-exponential decays it is recommended to use the amplitude weighted average lifetime ($< \tau >$) to calculate the transfer/quenching efficiency since $< \tau >$ is proportional to the steady-state intensity and the relative quantum yield.[S2] The amplitude weighted average lifetimes $< \tau >$ were calculated according to Equation 1,

$$< \tau >= \frac{\sum_i A_i \tau_i}{\sum_i A_i}, \tag{1}$$

where A_i is the amplitude for the $i$ths exponential component and τ_i is the corresponding lifetime. To study the trend of the radiative rate the intensity weighted averaged lifetime ($\bar{\tau}$) is more suitable. $\bar{\tau}$ is calculated according to Equation 2

$$\bar{\tau} = \frac{\sum_i A_i \tau_i^2}{\sum_i A_i \tau_i}, \tag{2}$$

From the photoluminescence quantum yield (Φ_{PL}) the average radiative decay constant k_r, and the non radiative rate constant (k_{nr}) can be calculated from Equation 3:

$$\Phi_{PL} = \frac{k_r}{k_r + k_{nr}} = k_r \bar{\tau}. \tag{3}$$

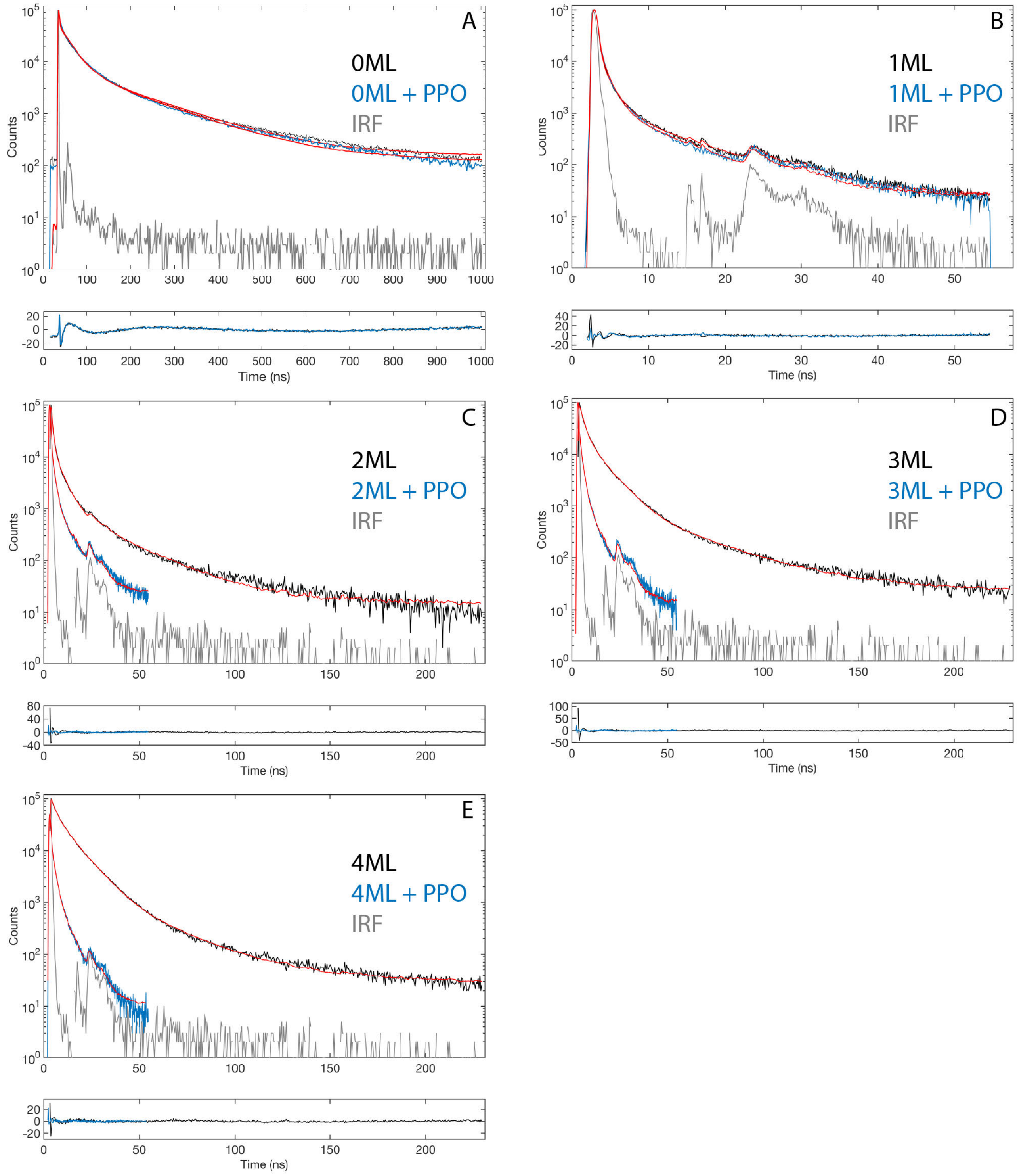

Figure S5: Photoluminescence decays of 3.55 nm diameter CdS core only nanocrysals (**0ML**, A) and CdS/ZnS core-shell nanocrystals with 1-4 monolayers (**1ML-4ML**, B-E) with (blue) and without (black) 5.7 mM 2,5-diphenyloxazole (PPO) in hexane. Shown is also the instrument response function (IRF) in grey and three exponential fits in red. Below each graph are the corresponding residuals. Decays are recorded at 415 nm after excitation at 406 nm.

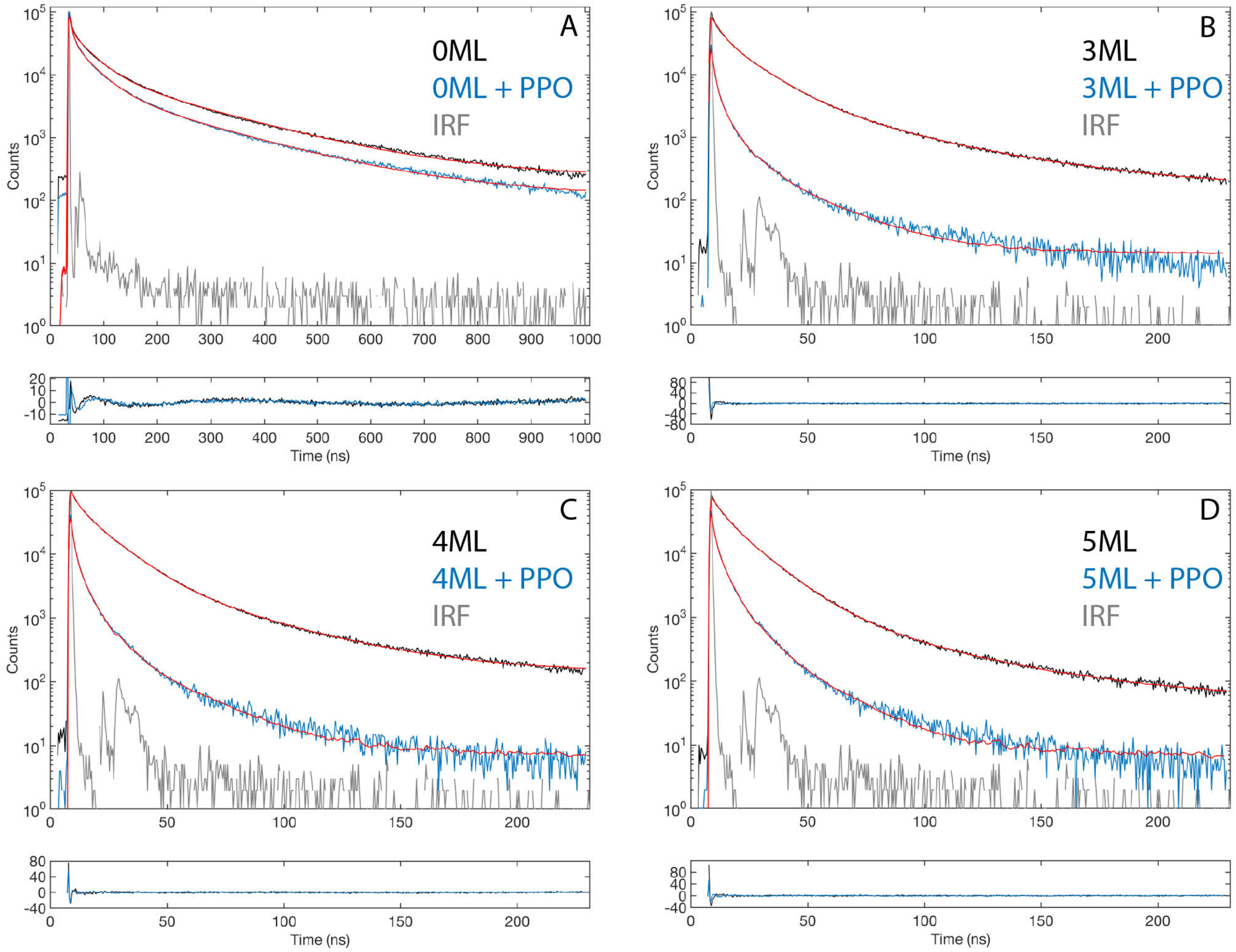

Figure S6: Photoluminescence decays of 4.3 nm diameter CdS core only nanocrysals (**0ML**, A) and CdS/ZnS core-shell nanocrystals with 3-5 monolayers (**1ML-4ML**, B-D) with (blue) and without (black) 5.7 mM 2,5-diphenyloxazole (PPO) in hexane. Shown is also the instrument response function (IRF) in grey and three exponential fits in red. Below each graph are the corresponding residuals. Decays are recorded at 430 nm after excitation at 406 nm.

Table S1: Obtained photoluminescence lifetimes (τ) and amplitudes (A from the fits in Figures S5-S6 and the intensity weighted average lifetime $\bar{\tau}$

NC	A_1	τ_1 (ns)	A_2	τ_2 (ns)	A_3	τ_3 (ns)	$\bar{\tau}$ (ns)
**0ML**[a]	0.65	1.11	0.06	141	0.29	25.9	83.2
**1ML**[a]	0.95	0.17	0.04	1.38	0.004	7.95	1.43
**2ML**[a]	0.87	0.70	0.13	4.36	0.008	23.3	5.32
**3ML**[a]	0.70	1.90	0.29	8.09	0.015	30.7	8.62
**4ML**[a]	0.46	1.84	0.51	8.41	0.025	25.6	9.35
**0ML**[b]	0.34	36.5	0.57	3.72	0.094	175	108
**3ML**[b]	0.46	14.4	0.06	47.5	0.48	3.95	21.0
**4ML**[b]	0.37	2.5	0.06	38.3	0.57	12.1	17.2
**5ML**[b]	0.38	4.42	0.03	42.2	0.59	12.9	15.1
**0ML** PPO[a]	0.63	1.21	0.06	137	0.31	26.2	-
**1ML** PPO[a]	0.94	0.15	0.06	1.11	0.004	6.53	-
**2ML** PPO[a]	0.88	0.29	0.12	1.31	0.008	6.09	-
**3ML** PPO[a]	0.81	0.30	0.18	1.26	0.014	4.76	-
**4ML** PPO[a]	0.77	0.38	0.20	1.52	0.025	4.98	-
**0ML** PPO[b]	0.30	37.2	0.64	5.88	0.064	173	-
**3ML** PPO[b]	0.20	5.21	0.024	22.3	0.77	1.15	-
**4ML** PPO[b]	0.78	1.11	0.011	21.9	0.21	5.17	-
**5ML** PPO[b]	0.68	1.13	0.022	17.9	0.30	5.06	-

[a] Core size 3.55 nm, [b]Core size 4.3 nm

Transient Absorption measurements

The transient absorption of CdS core only (**0ML**) with PPO (Figure S7) shows very similar features to **0ML** without PPO (Figure 5) indicating no interaction between the **0ML** CdS core NC and PPO.

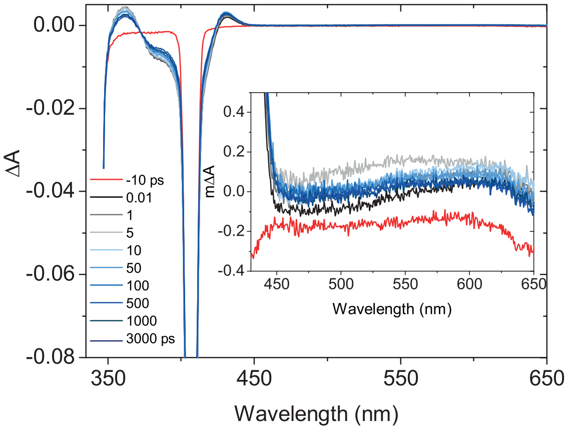

Figure S7: Transient absorption of nanocrystals (NCs) with 3.6 nm diameter CdS core and 2,5-diphenyloxazole (PPO). Inset shows the region 450-650 nm.

To verify that the measurements were performed in the linear regime without multi-photon annihilation occurring in the NCs the power dependence was measured. As seen in Figure S8 the NC's ground state bleach versus excitation power shows a linear trend in the whole excitation interval indicating that no multi-photon annihilation occurs at the excitation power used in our experiments.

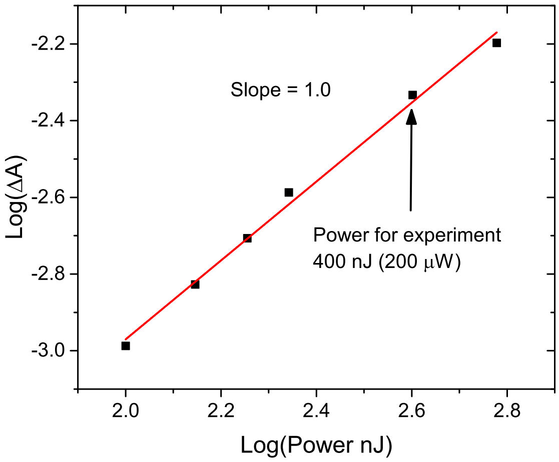

Figure S8: The intensity in the difference spectra of the CdS nanocrystal ground state bleaching (monitored at 432 nm) versus different excitation powers plotted in a log-log scale. Multi-photon annihilation was not observed in this excitation interval. Our experiments were performed in the linear region with a pump power of 400 nJ (200 μW).

References

(S1) Chen, D.; Zhao, F.; Qi, H.; Rutherford, M.; Peng, X. Bright and Stable Purple/Blue Emitting CdS/ZnS Core/Shell Nanocrystals Grown by Thermal Cycling Using a Single-Source Precursor. *Chem. Mater.* **2010**, *22*, 1437–1444.

(S2) Lakowicz, J. R. *Principles of Fluorescence Spectroscopy*, 3rd ed.; Springer Science: New York, 2006.
